# AKR1B10 regulates M2 macrophage polarization to promote the malignant phenotype of gastric cancer

**DOI:** 10.1042/BSR20222007

**Published:** 2023-09-28

**Authors:** Yi Wu, Yanjie Hao, Qing'xin Zhuang, Xiaoli Ma, Chao Shi

**Affiliations:** 1 Department of Medical Oncology, People’s Hospital of Ningxia Hui Autonomous Region, Yinchuan 750001, China; 2 Department of Medical Oncology, Sir Run Run Shaw Hospital, College of Medicine, Zhejiang University, Hangzhou 310000, Zhejiang, China; 3 Laser Department, People's Hospital of Ningxia Hui Autonomous Region, Yinchuan 750001, China; 4 Central lLaboratory, People’s Hospital of Ningxia Hui Autonomous Region, Yinchuan 750001, China

**Keywords:** AKR1B10, Immune infiltration, M2 macrophage polarization, WGCNA

## Abstract

Background: Immunotherapy has brought new hope to gastric cancer (GC) patients. Exploring the immune infiltration pattern in GC and the key molecules is critical for optimizing the efficacy of immunotherapy. Aldo-keto reductase family 1 member B10 (AKR1B10) is an inflammatory regulator and is closely related to the prognosis of patients with GC. However, the function of AKR1B10 in GC remains unclear.

Methods: In the present study, the CIBERSORT algorithm was used to analyze the immune infiltration pattern in 373 samples in the Cancer Genome Atlas (TCGA) database. Differentially expressed genes (DEGs) were seared by combing the TCGA database and the Gene Expression Omnibus (GEO) database, and the key molecule AKR1B10 was identified by weighted gene coexpression network analysis (WGCNA). The biological functions of AKR1B10 in stomach adenocarcinoma (STAD) were investigated *in vitro*.

Results: Macrophage polarization was the main immune infiltration pattern in GC, and the state of macrophage polarization was closely related to the pathological grading of GC and the clinical stage of patients. AKR1B10, MUC5AC, TFF2, GKN1, and PGC were significantly down-regulated in GC tissues. Low AKR1B10 expression induced M2 macrophage polarization and promoted the malignant phenotype of GC.

Conclusion: M2 macrophage polarization is the main immune infiltration pattern in GC. Low AKR1B10 expression induces M2 macrophage polarization and promotes the malignant transformation of GC.

## Introduction

Gastric cancer (GC) is the fifth most commonly diagnosed cancer, accounting for 8.2% of all cancer deaths, and is the third leading cause of cancer deaths worldwide [1]. Despite substantial improvements in diagnosis and treatment, the prognosis of GC patients is still poor [2]. In recent years, immunotherapy has brought new hope for the treatment of advanced GC. A global multicenter study, CHECKMATE-649 (CM649), found that the efficacy of chemotherapy combined with immunotherapy as a first-line treatment regimen for patients with human epidermal growth factor receptor 2-negative (HER-2−) advanced GC was superior to that of standard chemotherapy [3]. For patients with HER-2+ GC, the results of the KEYNOTE-811 study showed that the objective remission rate (ORR) after first-line treatment with pembrolizumab combined with trastuzumab + chemotherapy was as high as 74.4% [4]. Although the aforementioned large-scale phase III randomized controlled studies have basically established a role for immunotherapy in the first-line treatment of advanced GC, approximately 25–40% of patients still have disease progression after initial first-line treatment with immunotherapy combined with chemotherapy or immunotherapy combined with targeted therapy + chemotherapy. Exploring the immune infiltration pattern in GC and the key molecules is critical for optimizing the efficacy of immunotherapy.

In recent years, increasingly more studies have shown that the infiltration of immune cells plays a key role in the occurrence and development of cancers [5,6]. Additionally, there is also evidence that the composition of immune cells in the tumor microenvironment (TME) may affect the therapeutic effect and malignancy of GC [7,8]. As an analytical tool, CIBERSORT uses RNA-seq data to evaluate the expression of immune cells and obtain different proportions of immune cells. CIBERSORT has been widely used in the study of various TMEs, such as hepatocellular carcinoma (HCC) [9], colorectal cancer, Hodgkin’s lymphoma [10], and pancreatic cancer [11]. However, few studies have applied CIBERSORT to detect immune cell infiltration in GC.

Many recent studies have focused on the various effects of tumor-associated macrophages (TAMs) and found that macrophages have substantial effects on the TME [12]. TAMs are functionally heterogeneous and are divided into two major subpopulations, M1 and M2 macrophages [13]. M1 macrophages are the first line of defense against microbial infection. M1 macrophages maintain a strong antigen-presenting ability and induce a strong Th1 response. In contrast, M2 macrophages play a key role in limiting immune responses, inducing angiogenesis, and repairing tissue. Therefore, the presence of M2 TAMs is associated with tumor-promoting activity, and the presence of M1 TAMs is associated with antitumor activity. These two phenotypes represent the extremes of TAM functions, and the M2 phenotype is greatly related to the initiation, development, progression, and poor prognosis of cancers [14]. The cytokines present in the TME enable TAMs to achieve a tumorigenic phenotype (M2 macrophages) and exert immunosuppressive effects [15,16]. In general, TAMs are highly plastic cells, and their phenotypic reprogramming of antitumor responses has been the focus of current research.

The aldo-keto reductase (AKR) protein superfamily contains more than 190 members and is divided into 16 families, which are present in all plant flora. These enzymes reduce carbonyl substrates, such as peroxide byproducts of sugar aldehydes, ketone steroids, ketone prostaglandins, retinoids, quinones, and lipids. Aldo-keto reductase family 1 member B10 (AKR1B10) is located at 7q33 and was originally thought to be a factor that regulates retinoic acid signaling by converting all-trans-retinal to retinol [17]. It is highly overexpressed in the development of non-small cell lung cancer and liver cancer and is considered to be a tumor marker [17,18]. Furthermore, AKR1B10 has been identified as a regulator of inflammation [19]. It is required for the nuclear translocation of nuclear factor kappa B (NF-κB) and the phosphorylation/degradation of IκB-α and stimulates the expression of proinflammatory cytokines [20]. In addition, AKR1B10 is expressed in various tissues of the digestive tract, such as the stomach, small intestine, and colorectal region, but is down-regulated in gastrointestinal cancer and inflammatory bowel disease [21]. Studies have confirmed that compared with that in GC patients without lymph node metastasis, AKR1B10 was significantly reduced in GC patients with lymph node metastasis; furthermore, it was negatively correlated with tumor size (*P*<0.001), depth of invasion (*P*<0.001), and tumor, nodes, and metastasis (TNM) stage (*P*<0.001) and regulated the epithelial–mesenchymal transition (EMT) of GC cells [22]. Limited by the complexity of the TME, the effect of AKR1B10 on GC cells is not sufficient to explain the important role of this gene in the development and progression of cancers. Exploring the role of AKR1B10 in the immune microenvironment of GC is critical to determining whether AKR1B10 can become a potential target for tumor treatment.

## Method

### Quantification of tumor-infiltrating immune cells (TIICs) using the CIBERSOFT algorithm

The CIBERSORT algorithm was used to determine the expression distribution of 547 immune genes in various types of immune cells and to quantify the proportion of each type of immune cell. This method was used to infer the relative proportion of study samples among the 22 types of immune cells. The gene expression dataset was prepared using standard annotation files, and the data were uploaded to the CIBERSORT network and run using the default signature matrix and 1000 permutations. Using the CIBERSORT algorithm, the *P-*value was obtained for each deconvoluted sample through Monte Carlo sampling, confirming that each result is credible.

### Weighted Gene Coexpression Network Analysis (WGCNA)

All samples in the Cancer Genome Atlas (TCGA) and GSE54129 were included in the WGCNA (‘WGCNA’ and related software packages in R) to locate the gene modules that are coexpressed and to explore the association between the gene network and the phenotype of interest, as well as the hub genes in the network.

### Functional and pathway enrichment analysis

Gene ontology (GO) [18] and Kyoto Encyclopedia of Genes and Genomes (KEGG) (http://www.genome.ad.jp/kegg/) [19] are the most commonly used tools to describe molecular biological information, such as gene functions, biological functions, protein networks, and genomic information. The functional and pathway enrichment analysis was performed using ‘ClusterProfiler’ in the R software package, and the potential biological significance of the target gene set in each subtype was revealed. The cut-off points were |log2-fold-change|≥0.2 and *P*<0.05.

### Cell culture

Human GC cell lines (HGC-27 and AGS) and mononuclear THP-1 cells (American Type Culture Collection, VA, U.S.A.) were cultured in Dulbecco’s Modified Eagle Medium (DMEM) containing 10% fetal bovine serum (both from Thermo Fisher Scientific, MA, U.S.A.). To generate M0 macrophages (M0), THP-1 cells were treated with 100 ng/ml phorbol 12-myristate 13-acetate (PMA, AdipoGen, San Diego, CA, U.S.A.) for 24 h. Once differentiated, they were incubated with IL-4 and IL-13 in order to obtain M2 polarized macrophages or with IFN-gamma and LPS for classical macrophage activation (M1).

### Transient transfection

Macrophages were transiently transfected. AKR1B10-short hairpin (sh)RNA and related NCs were purchased from Genechem Co., Ltd. (Shanghai, China). Macrophages were seeded into six-well plates at a density of 4 × 10^5^ cells/ml and cultured. When the cells reached a confluence of 80%, they were transfected following the instructions provided with the lipofectamine 3000 reagent (Invitrogen, CA, U.S.A.). After transfection, the cells were cultured for 48 h for subsequent experiments.

### Quantitative real-time reverse transcription PCR

Total RNA was extracted from the cells using TRIzol reagent (Invitrogen). Reverse transcription was performed using a reverse transcription kit (TAKARA) following the manufacturer’s protocol. Real-time PCR was performed using a standard SYBR Green PCR kit (Qiagen, Germany). Glyceraldehyde-3-phosphate dehydrogenase (GAPDH) was used as an internal reference. The sequences of the different primers used are provided in Table 1. Each sample was analyzed in triplicate, and the relative quantification of gene expression was determined using the 2-^△△CT^ method.

**Table 1:** Sequence of primers used in the study

Genes	Forward sequence	Reverse sequence
U6	F: 5′-ATTGGAACGATACAGAGAAGATT-3′	R: 5′-GGAACGCTTCACGAATTTG-3′
GAPDH	F: 5′-ACGGCAAGTTCAACGGCACAG-3′	R: 5′-GACGCCAGTAGACTCCACGACA-3′
CD206	F: 5′-GGGTTGCTATCAC TCTCTATGC-3′	R: 5′-TTTCTTGTCTGTTGCCGTAGTT-3′
CD163	F: 5′-ACATAGATCATGCATCTGTCATTTG-3′	R: 5′-CATTCTCCTTGGAATCTCACTTCTA-3′
CCL17	F: 5′-CAGGAAGTTGGTGAGCTGGTA-3′	R: 5′-TTGTGTTCGCCTGTAGTGCATA-3′
CCL18	F: 5′-TGGCAGATTCCACAAAAGTTCA-3′	R: 5′-GGATGACACCTGGCTTGGG-3′
AKR1B10	F: 5′-TCAGAATGAACATGAAGTGGGG-3′	R: 5′-TGGGCCACAACTTGCTGAC-3′
TNF-α	F: 5′-CCTCTCTCTAATCAGCCCTCTG-3′	R: 5′-GAGGACCTGGGAGTAGATGAG-3′
IL-6	F: 5′-ACTCACCTCTTCAGAACGAATTG-3′	R: 5′-CCATCTTTGGAAGGTTCAGGTTG-3′
TGF-β	F: 5′-GGCCAGATCCTGTCCAAGC-3′	R: 5′-GTGGGTTTCCACCATTAGCAC-3′

### Coculture of THP-1 and GC cells

THP-1 macrophages were divided into two groups (control group and sh-AKR1B10 group) for different treatments. After 48 h of incubation, the medium was replaced with fresh medium, and the incubation was continued for 24 h. Then, the supernatant was collected and used as the macrophage culture medium for the culture of HGC-27 and AGS GC cells.

### Migration assay

GC cells cocultured with macrophage supernatant were seeded into the upper chamber of a 24-well Transwell plate (8.0 μm; Corning, NY, U.S.A.), and 500 μl of DMEM containing 10% FBS was placed in the lower chamber. Then, the Transwell plates were incubated in a 37°C, 5% CO_2_ incubator for 12 h. The cells were then fixed with 4% paraformaldehyde for 10 min and stained with 0.01% Crystal Violet. The cells that had not migrated were carefully removed with a cotton swab, and the cells that had migrated to the lower chamber were counted under a microscope.

### Cell counting kit-8 (CCK8)

GC cells cocultured with macrophage supernatant were seeded into 96-well plates (2 × 10^3^ cells/well). Afterwards, cell viability was assessed using the Cell Counting Kit-8 (CCK-8; Dojindo Molecular Technology Co., Ltd., Japan) at 24, 48, 72, and 96 h following the manufacturer’s instructions.

### Colony formation experiment

GC cells cocultured with macrophage supernatant were seeded into six-well plates (1 × 10^3^ cells/well) and cultured for 1 week. The cells in the six-well plate were fixed with 4% paraformaldehyde for 15 min, stained with Crystal Violet for 10 min, and photographed and counted.

### Statistical analysis

SPSS13.0 (SPSS, U.S.A.) was used for the data analysis. Data are expressed as the mean ± standard deviation. Comparisons between two groups were performed using the independent sample *t*-test, and comparisons among multiple groups were performed using one-way analysis of variance (ANOVA). *P*<0.05 was defined as a significant difference.

## Results

### CIBERSORT analysis indicates that macrophage polarization is the main immune infiltration pattern in GC

**Figure 1:**
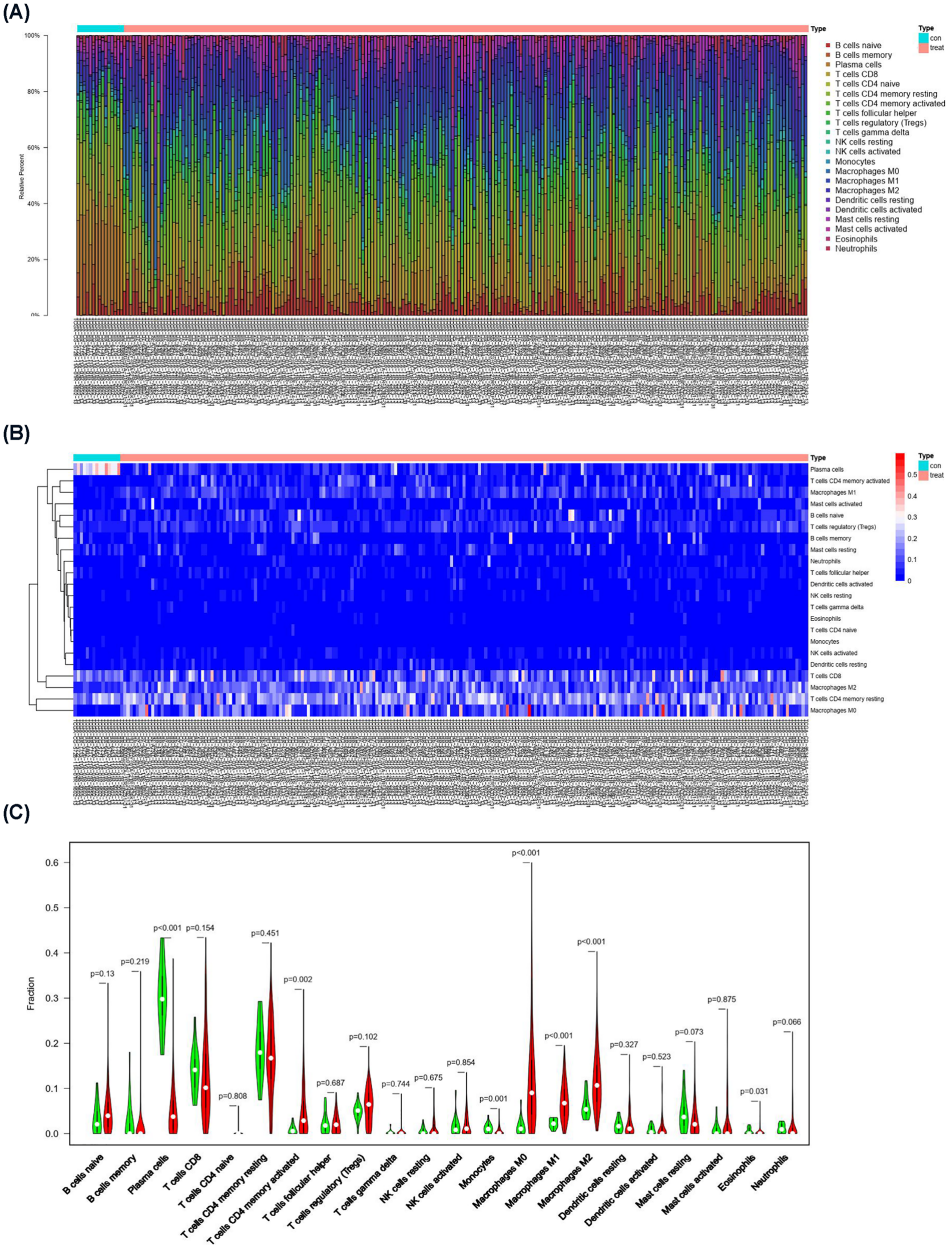
CIBERSORT analysis revealed that M2 macrophage polarization was the main immune infiltration pattern in GC (**A**) Bar graph showing the proportion of 22 types of immune cell infiltration in GC-adjacent tissue and tumor tissue. (**B**) Heat map showing the differences in the distribution of 22 types of immune cell infiltration in GC-adjacent tissue and tumor tissue. (**C**) Piano key diagram showing the differences in 22 types of immune cell components in GC-adjacent tissues and tumor tissues.

To investigate the proportion of various immune cells in GC samples, we analyzed and compared 22 types of immune cells in 30 normal tissues and 343 GC tissues in the TCGA database using the CIBERSORT algorithm. Figure 1A shows the proportion of various types of immune cell infiltration in the 373 samples. The 30 samples in the left panel are normal tissue, and the 343 samples in the right panel are tumor tissue. There was a significant difference in the immune cell infiltration pattern between normal tissue and tumor tissue. The heat map of immune cell infiltration in these samples (Figure 1B) indicated that the distribution of some immune cells in normal tissues and tumor tissues was significantly different. Plasma cells accounted for a higher proportion of immune cells in normal tissues, and M2 macrophages accounted for a higher proportion of immune cells in tumor tissues. Comparisons of the proportions of various types of immune cells in normal tissue and tumor tissue showed that the proportions of M0 macrophages (*P*<0.001), M1 macrophages (*P*<0.001), and M2 macrophages (*P*<0.001) were relatively high in tumor tissue (Figure 1C). These results indicate that macrophage polarization is the main immune infiltration pattern in GC.

### The polarization state of macrophages is closely related to the clinical characteristics of GC

**Figure 2:**
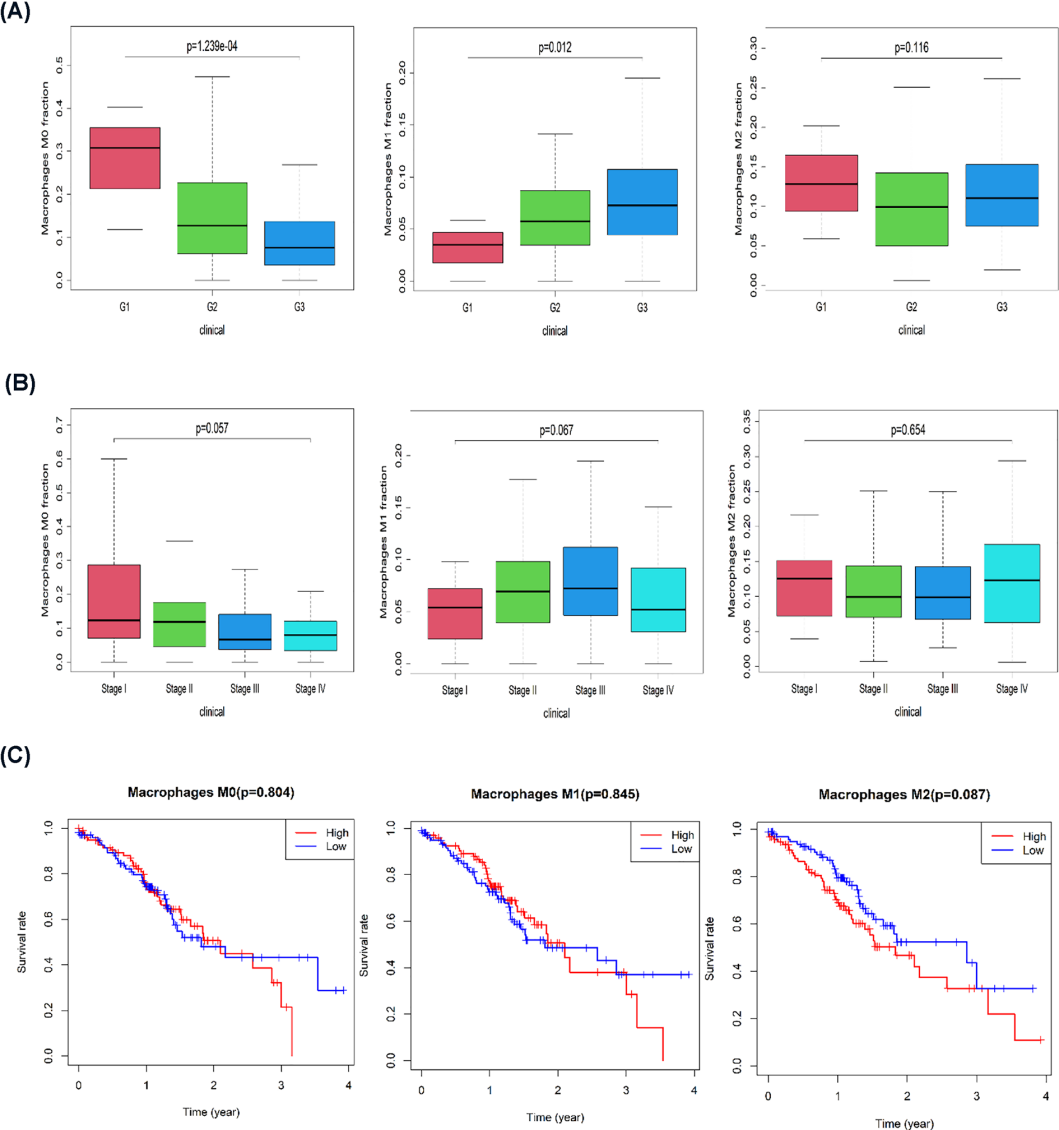
The polarization state of macrophages was closely related to the clinical characteristics of GC (**A**) Histogram showing the proportions of M0, M1, and M2 macrophages among different pathological grades. (**B**) Histogram showing the proportions of M0, M1, and M2 macrophages among different clinical stages. (**C**) Kaplan–Meier survival curve analysis of the relationship between the levels of M0, M1, and M2 macrophages and patient prognosis.

In GC patients with different pathological tumor grades and in different clinical stages, macrophages are in different polarization states. An analysis of the clinical characteristics of GC patients in the TCGA database showed that with an increase in the degree of malignancy, M0 macrophage infiltration gradually decreased, the proportion of M1 macrophage infiltration increased, and the proportions of M2 macrophages in different pathological grades was different (Figure 2A). With the progression of tumor stages, the proportion of M0 macrophages gradually decreased, and the proportion of M1 macrophages increased in stages 1–3 and decreased in stage 4. There was no significant difference in M2 macrophages between different stages (Figure 2B). A survival analysis indicated that M0 macrophage content and low and high M1 macrophage content had no significant effect on GC patient survival but that low M2 macrophage content was associated with a better prognosis than was high M2 macrophage content (Figure 2C). Therefore, the polarization state of macrophages is closely related to the clinical characteristics of GC.

### Differentially expressed genes (DEGs) in tumor patients and nontumor patients

**Figure 3:**
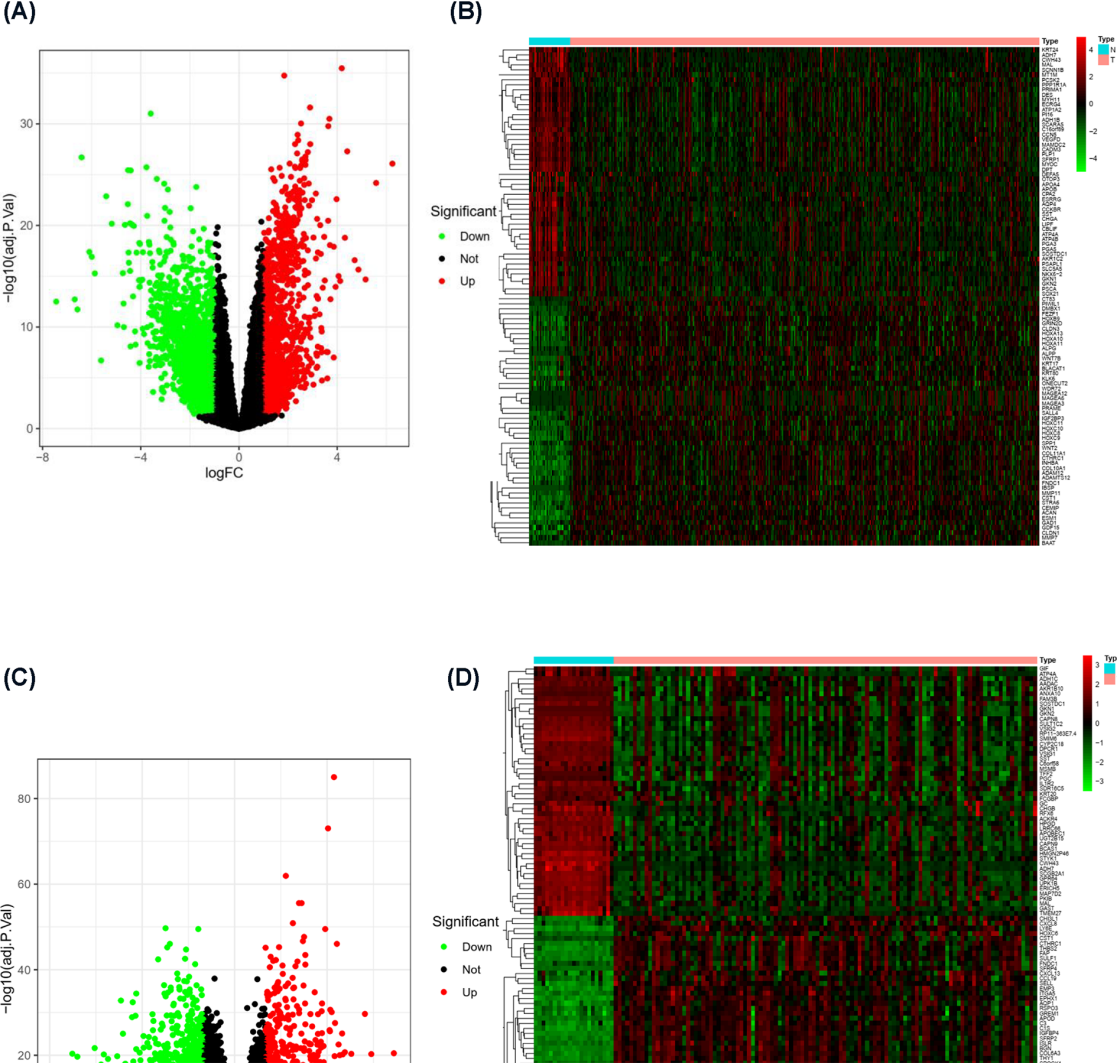
DEGs in tumor tissue and nontumor tissue (**A**) Volcano plot of DEGs in the TCGA database (log FC > 1). (**B**) Heat map of the top 50 DEGs in the TCGA database. (**C**) Volcano plot of DEGs in GSE54129 (log FC > 1). (**D**) Heat map of the top 50 DEGs in GSE54129.

The polarization status of macrophages was significantly different between tumor tissue and normal tissue. To further search for the key molecules that affect the polarization status of macrophages, we combined the TCGA database and the Gene Expression Omnibus (GEO) database to search for DEGs. In the analysis of 30 normal tissue samples and 343 GC tissue samples in the TCGA database, a total of 1122 up-regulated genes and 1563 down-regulated genes (absolute value of log FC > 1) in tumors were identified (Figure 3A). A heat map of the top 50 DEGs between the two groups is shown in Figure 3B. In addition, we also analyzed the GEO dataset GSE54129. A total of 21 normal tissue samples and 111 GC tissue samples were included. Using absolute value of log FC > 1, 895 up-regulated genes and 899 down-regulated genes in tumors were identified (Figure 3C). A heat map of the top 50 DEGs between two groups in this database is shown in Figure 3D. Through the analysis of the above two authoritative data sets, we preliminarily identified DEG sets for GC patients and normal patients.

### WGCNA identified a risk score-related module in patients with stomach adenocarcinoma (STAD)

**Figure 4:**
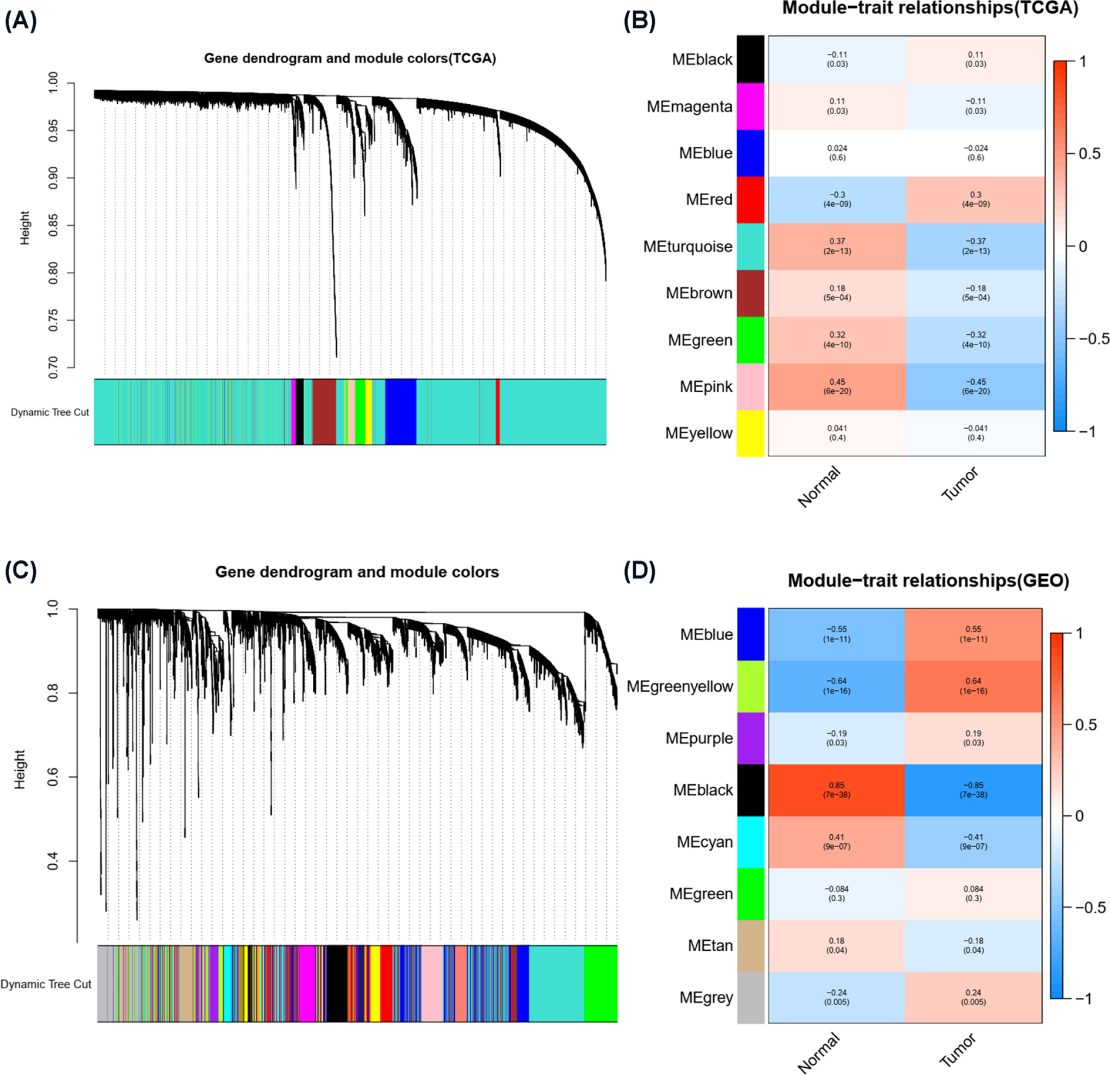
The risk score-related modules of STAD patients were determined by WGCNA (**A**) Clustering dendrogram of the coexpression network module from the TCGA database. (**B**) Heat map of the association analysis between the modules and traits from the TCGA database. Blue indicates a negative correlation, and red indicates a positive correlation. The *P-*value is shown in the figure. (**C**) Clustering dendrogram of the coexpression network module from the GSE54129 database. (**D**) The heat map of the association analysis between the modules and traits from the GSE54129 database. Blue indicates a negative correlation, and red indicates a positive correlation. The *P-*value is shown in the figure.

To further improve the accuracy of the analysis to find hub genes, we performed WGCNA of the above two databases. The WGCNA algorithm clusters genes with similar expression into a module by calculating correlations between the expression of different genes and then analyzes the correlation between the module and the disease phenotype to screen hub genes. A hierarchical clustering tree was obtained for samples in the TCGA database using the WGCNA algorithm (Figure 4A). In the tree, each leaf represents a gene. Genes with similar expression data form branches of the tree, and each color represents a gene module. Several modules were generated, and the correlation between each module and the disease phenotype is shown in Figure 4B. The MEpink module in Figure 4B was selected as the most positively correlated with normal tissue (correlation coefficient = 0.69, *P*=2e-52) and the most negatively correlated with tumor tissue. Similarly, GSE54129 (111 GC tissue samples and 21normal tissue samples) was analyzed using the WGCNA algorithm, and dendrograms (Figure 4C) and module diagrams (Figure 4D) were obtained. The MEblack module was selected as the most positively correlated with normal tissue (correlation coefficient = 0.69, *P*=2e-52) and the most negatively correlated with tumor tissue.

### STAD hub genes and analysis

**Figure 5:**
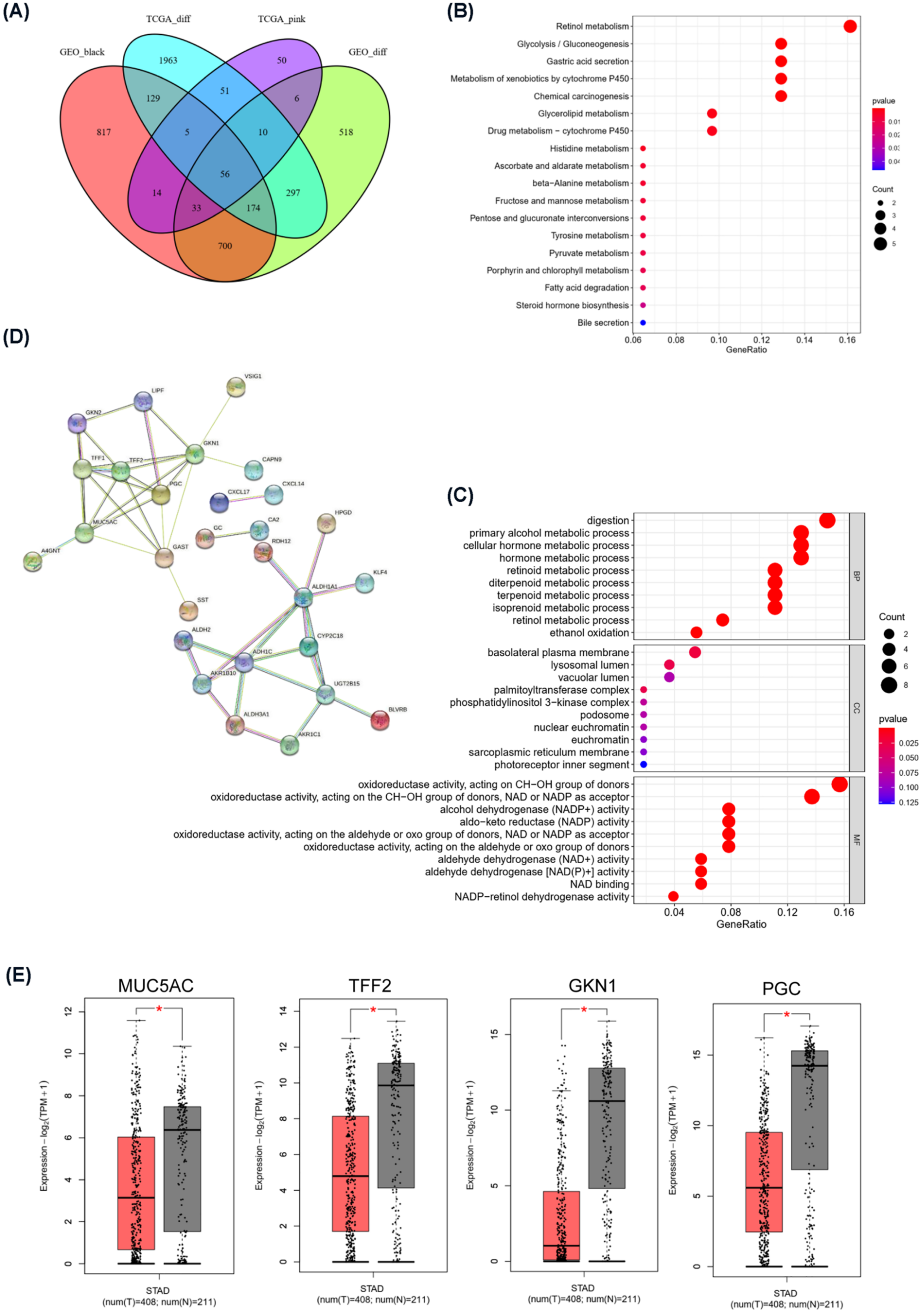
STAD hub genes and analysis (**A**) Common down-regulated protective genes were screened using a Venn diagram. (**B**) KEGG pathway enrichment analysis of protective genes. The *X*-axis represents the *P-*value, and the size of the bubble represents the number of protective genes. The *Y*-axis represents the terms of the significantly enriched pathways. (**C**) GO terms with significant enrichment of protective genes. The *X*-axis represents the adjusted *P-*value, and the size of the bubble represents the number of DEGs involved. The *Y*-axis represents the terms of significantly enriched functions. (**D**) Protein–protein interaction (PPI) network constructed with the protective genes in the STRING database. (**E**) Expression of hub genes (MUC5AC, TFF2, GKN1, and PGC) in STAD (Note: Red represents tumor tissue, and gray represents normal tissue). *P<0.01.

To further determine the hub genes associated with tumors, we took the intersection of the DEGs from the TCGA and GEO databases and the most significant modular genes obtained from the WGCNA, resulting in 56 genes (Figure 5A). These genes were down-regulated in tumor tissue and up-regulated in normal tissues and are considered to be protective genes that have inhibitory effects on tumors. KEGG analysis showed that the pathways these genes involved in are concentrated in retinol metabolism (*P*<0.05), glycolysis/gluconeogenesis (*P*<0.05), and gastric acid secretion (*P*<0.05) (Figure 5B). GO analysis indicated that these genes were mainly involved in the digestion process, alcohol metabolism, and the regulation of oxidoreductase activity (Figure 5C). To further explore the relationship and interaction among these genes, we analyzed the interaction relationships among the proteins encoded by these genes through STRING (Figure 5D). The node molecules MUC5AC, TFF2, GKN1, and PGC at the center of the protein–protein interaction network are considered to be the hub genes of STAD, and their expression levels in GC tissues were all significantly lower than those in normal gastric mucosal tissues (Figure 5E).

### AKR1B10 deletion induces M2 macrophage polarization in GC

**Figure 6:**
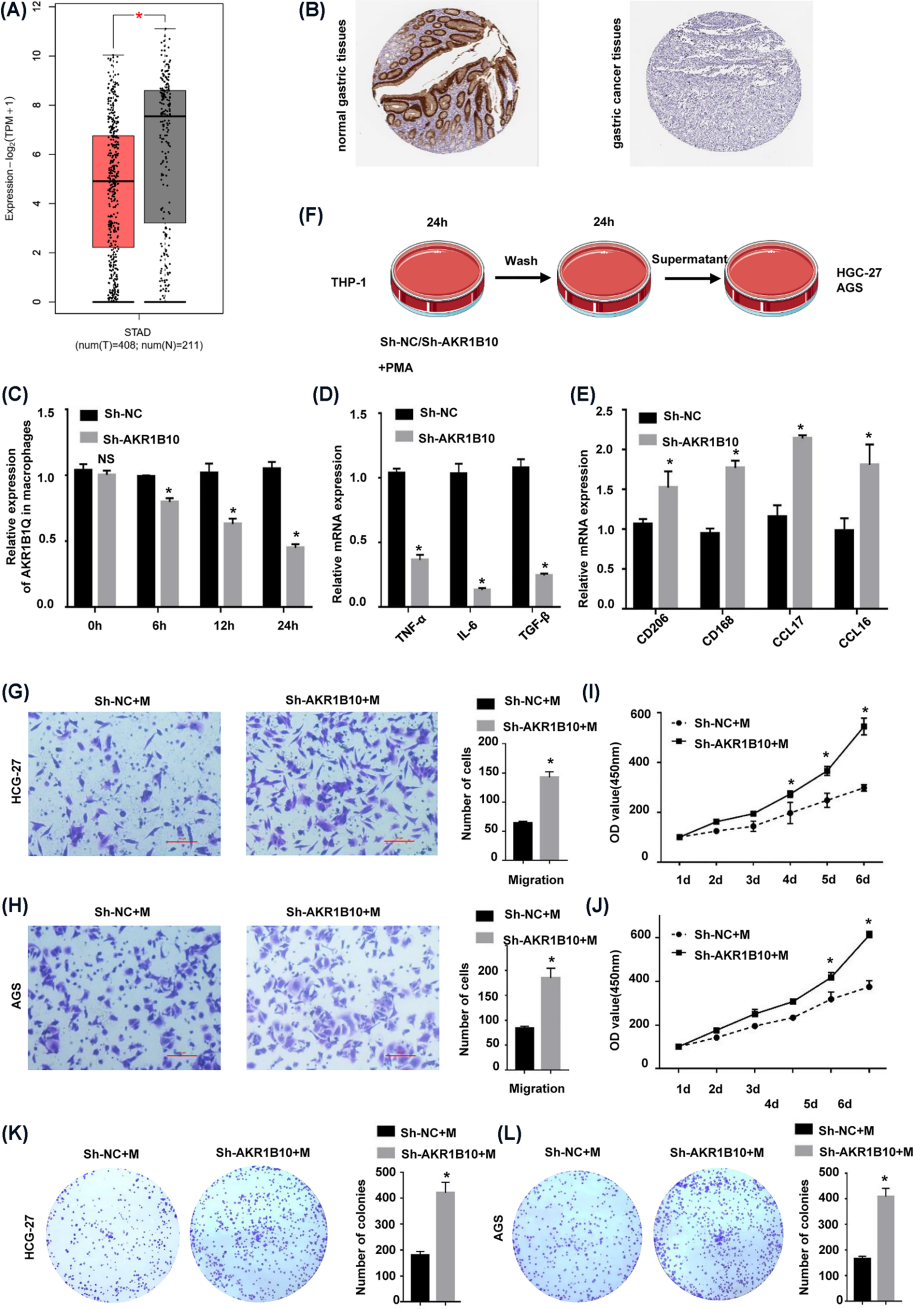
Down-regulation of AKR1B10 induced M2 macrophage polarization in GC (**A**) Expression of AKR1B10 in STAD. (Note: Red represents tumor tissue, and gray represents normal tissue.) (**B**) Immunohistochemical analysis of AKR1B10 protein expression in STAD tissue and normal tissue. (**C**) Detection of AKR1B10 expression in macrophages by RT-qPCR. (**D**) RT-qPCR detection of M2 macrophage markers (CD206, CD163, CCL17 and CCL18). (**E**) RT-qPCR detection of M1 macrophage markers (TNF-α, IL-6 and TGF-β). (**F**) The coculture model diagram of macrophages and gastric cancer cells. (**G,H**) Transwell results for HGC-27 and AGS GC cells cocultured with macrophages. (**I,J**) CCK-8 results for HGC-27 and AGS GC cells cocultured with macrophages. (**K,L**) Colony formation for HGC-27 and AGS GC cells cocultured with macrophages. *P<0.01.

AKR1B10 was significantly down-regulated in GC samples, and its expression was higher in normal stomach tissue (Figure 6A). Immunohistochemical analysis showed that AKR1B10 protein was mainly expressed around the gastric glands, normal glandular structure was destroyed in GC sections, and AKR1B10 expression was extremely low (Figure 6B). Affected by the TME, macrophages play an important role in the development and progression of GC. Studies have shown that AKR1B10, as a chemokine, can stimulate macrophages to polarize in different directions. The treatment of THP-1 macrophages with sh-AKR1B10 showed that the expression of AKR1B10 in macrophages decreased after 24 h (Figure 6C). Compared with the control group, THP-1 macrophages treated with sh-AKR1B10 showed a decrease in the M1 polarization markers TNF-α, IL-6, and TGF-β after LPS stimulation (Figure 6D) and an increase in the M2 polarization markers CD206, CD163, CCL17, and CCL18 after PMA stimulation (Figure 6E), indicating that AKR1B10 deletion induced a pro-oncogenic phenotype in macrophages. The supernatant of THP-1 macrophages treated with sh-AKR1B10 or sh-NC was cocultured with HGC-27 and AGS GC cells for 24 h (Figure 6F). The GC cells cultured with supernatant from THP-1 macrophages with down-regulated AKR1B10 expression exhibited increased migration (Figure 6G,H) and proliferation capacities (Figure 6I–L). These results indicate that the down-regulation of AKR1B10 expression induces macrophages polarization in the direction of promoting cancers, thereby promoting the proliferation and metastasis of GC.

## Discussion

GC is a common cancer worldwide. Although GC research has made gradual progress, the underlying molecular mechanism of GC is still unclear [23]. In the present study, we first used CIBERSORT to analyze the immune infiltration patterns in 343 GC tissues and 30 normal tissues and found that M2 macrophage polarization is an important immunological feature of GC tissue. In addition, with disease progression and pathological deterioration, the polarization state of macrophages is closely related to the clinical characteristics of GC patients. Bioinformatics analysis of the genetic information of GC patients in the TCGA database and the GEO database showed that AKR1B10, MUC5AC, TFF2, GKN1, and PGC expression was significantly different between GC tissue and normal tissue and that AKR1B10 expression was closely related to the polarization state of macrophages. After AKR1B10 inhibition in macrophages, M2 macrophage polarization was activated to promote the proliferation and metastasis of GC cells.

Clinical studies have strongly demonstrated that macrophages can promote tumorigenesis. In a meta-analysis, it was reported that >80% of the studies showed an association between macrophage density and poor patient prognosis [24]. In lung cancer and liver cancer, poor survival rates are associated with increased macrophage density [25,26]. Classically activated (M1) macrophages exhibit tumor-killing activity after exposure to interferon γ (IFNγ) and cause tissue destructive reactions. When stimulated by IL-4 or IL-13, macrophages undergo alternative (M2) activation [27]. In general, M2 cells acquired in response to IL-4 are oriented toward tissue repair and remodeling, immune regulation, and tumor promotion [28,29]. In the initial stage of tumor formation, macrophages create a mutagenic inflammatory environment, and the phenotype of macrophages changes from an ‘inflammatory’ type to one that resembles macrophages that promote tissue formation during development [30,31]. When tumors progress to malignancy, macrophages stimulate angiogenesis [32,33]. Macrophages can produce proteases that breakdown the extracellular matrix [34–36] and enhance the migration and invasion of tumor cells. Our analysis of the TCGA database showed that the infiltration of M2 macrophages was significantly higher in GC tissue and that the polarization state of macrophages was closely related to the clinical characteristics of GC. M2 macrophages also release cytokines and chemokines such as IL-6, TNFα, and CCL2 [37], activate the TGF-β signaling pathway [38], and promote EMT [39], thereby promoting the occurrence and development of tumors.

AKR1B10 expression was significantly reduced in GC, and AKR1B10 mRNA expression was reduced by at least 50-fold in 45.8% of GC tumors. Correspondingly, AKR1B10-positive GC specimens were more frequently from patients with a tumor size <5 cm, no lymph node metastasis, no distant metastasis, and a low tumor stage. Compared with that for the AKR1B1-negative group, the 5-year survival rate for the AKR1B10-positive group was significantly higher than that for the AKR1B1-negative group, a finding that is consistent with a previous study [40]. Studies have found that the reduction in AKR1B10 expression promotes the proliferation and migration of GC cells. In addition, AKR1B10 is significantly associated with EMT, and AKR1B10 knockout significantly increases vimentin and E-cadherin expression [41]. AKR1B10 is a member of the AKR superfamily. It is a nicotinamide adenine dinucleotide phosphate-dependent oxidoreductase on the cell membrane that can metabolize carbohydrates, steroids, prostaglandins, and exogenous carbonyl compounds [42]. The expression of AKR1B10 is mainly limited to the distal gastrointestinal tract, including the small intestine and colon [43]. Clinical pathological studies have shown that the expression of AKR1B10 in HCC varies with disease stage. AKR1B10 was significantly overexpressed in the early stage of diseases such as cirrhosis or viral hepatitis and down-regulated in the advanced stage of poorly differentiated tumors [44,45]. This indicates that AKR1B10 may change dynamically during the progression of HCC and may also play a role in the early stage of HCC development. In future studies, the function and mechanism of AKR1B10 should be explored through gene knockout or overexpression models in mouse macrophages. AKR1B10 inactivation is caused by the specific regulation of oncogenic transcription factors. Studies have found that AKR1B10 contains several putative regulatory motifs, such as AP-1, NF-κB, and antioxidant response elements. The transcription factor Nrf2 is one of the main factors involved in the regulation of AKR1B10 gene [46]. In addition, AKR1B10 is a key enzyme involved in the expression of proinflammatory cytokines. The analysis of transcriptome data of lung samples from coronavirus disease 2019 (COVID-19) patients showed that the expression of the gene encoding AKR1B10 was increased. In macrophages and lung cells, AKR1B10 overexpression induces the expression of the proinflammatory cytokines interleukin-6 (IL-6), IL-1β and tumor necrosis factor α (TNFα) [47]. The evidence that AKR1B10 is involved in inflammatory responses has been confirmed in diabetic nephropathy (DN). Under high glucose conditions and LPS stimulation, AKR1B10 expression in peripheral blood mononuclear cells (PBMCs) of DN patients was significantly increased compared with that in those without DN and a normal control group [48].

## Data Availability

Our study used public online database. The data can be accessed by following websites: https://www.cancer.gov/about-nci/organization/ccg/research/structural-genomics/tcga; https://www.ncbi.nlm.nih.gov/geo/query/acc.cgi?acc=GSE54129

## Abbreviations

AKR: the aldo-keto reductase
AKR1B10: aldo-keto reductase family 1 member B10
AP-1: activator protein-1
CCL17: C-C motif chemokine ligand 17
CCL18: C-C motif chemokine ligand 18
CCL2: C-C motif chemokine ligand 2
CD163: CD163 molecule
CD206: CD206 molecule
DGE: Differentially expressed gene
DN: diabetic nephropathy
EMT: epithelial–mesenchymal transition
FC: fold change
GAPDH: glyceraldehyde-3-phosphate dehydrogenase
GC: gastric cancer
GKN1: gastrokine 1
IFNγ: interferon-γ
IL-13: interleukin-13
IL-1β: interleukin-1β
IL-4: interleukin-4
IL-6: interleukin-6
IκB-α: inhibitor of κB
LPS: lipopolysaccharide
MUC5AC: Mucin 5AC, Oligomeric Mucus/Gel-Forming
NF-κB: nuclear factor kappa B
Nrf2: nuclear factor E2-related factor 2
ORR: objective remission rate
PBMC: peripheral blood mononuclear cell
PGC: progastricsin
PMA: phorbol 12-myristate 13-acetate
STAD: stomach adenocarcinoma
TAM: tumor-associated macrophage
TFF2: Trefoil Factor 2
TGF-β: transforming growth factor β
TME: tumor microenvironment
TNFα: tumor necrosis factor-α
